# The South Atlantic Dipole via multichannel singular spectrum analysis

**DOI:** 10.1038/s41598-024-62089-w

**Published:** 2024-07-05

**Authors:** Gaston Manta, Eviatar Bach, Stefanie Talento, Marcelo Barreiro, Sabrina Speich, Michael Ghil

**Affiliations:** 1 Geosciences Department and Laboratoire de Météorologie Dynamique (CNRS and IPSL), École Normale Supérieure and PSL University, Paris, France; 2 Department of Atmospheric Sciences and Ocean Physics, Facultad de Ciencias, Universidad de la República, Montevideo, Uruguay; 3 Scripps Institution of Oceanography, La Jolla, CA USA; 4 Department of Environmental Science and Engineering and Department of Computing and Mathematical Sciences, California Institute of Technology, Pasadena, CA USA; 5 Potsdam Institute for Climate Impact Research, Potsdam, Germany; 6 Department of Atmospheric and Oceanic Sciences, University of California at Los Angeles, Los Angeles, CA USA; 7 Department of Mathematics, Imperial College London, London, USA; 8 Department of Meteorology and Department of Mathematics and Statistics, University of Reading, Reading, United Kingdom

**Keywords:** Atmospheric science, Climate change, Ocean sciences

## Abstract

This study analyzes coupled atmosphere–ocean variability in the South Atlantic Ocean. To do so, we characterize the spatio-temporal variability of annual mean sea-surface temperature (SST) and sea-level pressure (SLP) using Multichannel Singular Spectrum Analysis (M-SSA). We applied M-SSA to ERA5 reanalysis data (1959–2022) of South Atlantic SST and SLP, both individually and jointly, and identified a nonlinear trend, as well as two climate oscillations. The leading oscillation, with a period of 13 years, consists of a basin-wide southwest–northeast dipole and is observed both in the individual variables and in the coupled analysis. This mode is reminiscent of the already known South Atlantic Dipole, and it is probably related to the Pacific Decadal Oscillation and to El Niño–Southern Oscillation in the Pacific Ocean. The second oscillation has a 5-year period and also displays a dipolar structure. The main difference between the spatial structure of the decadal, 13-year, and the interannual, 5-year mode is that, in the first one, the SST cold tongue region in the southeast Atlantic’s Cape Basin is included in the pole closer to the equator. Together, these two oscillatory modes, along with the trend, capture almost 40% of the total interannual variability of the SST and SLP fields, and of their co-variability. These results provide further insights into the spatio-temporal evolution of SST and SLP variability in the South Atlantic, in particular as it relates to the South Atlantic Dipole and its predictability.

## Introduction

**Figure 1:**
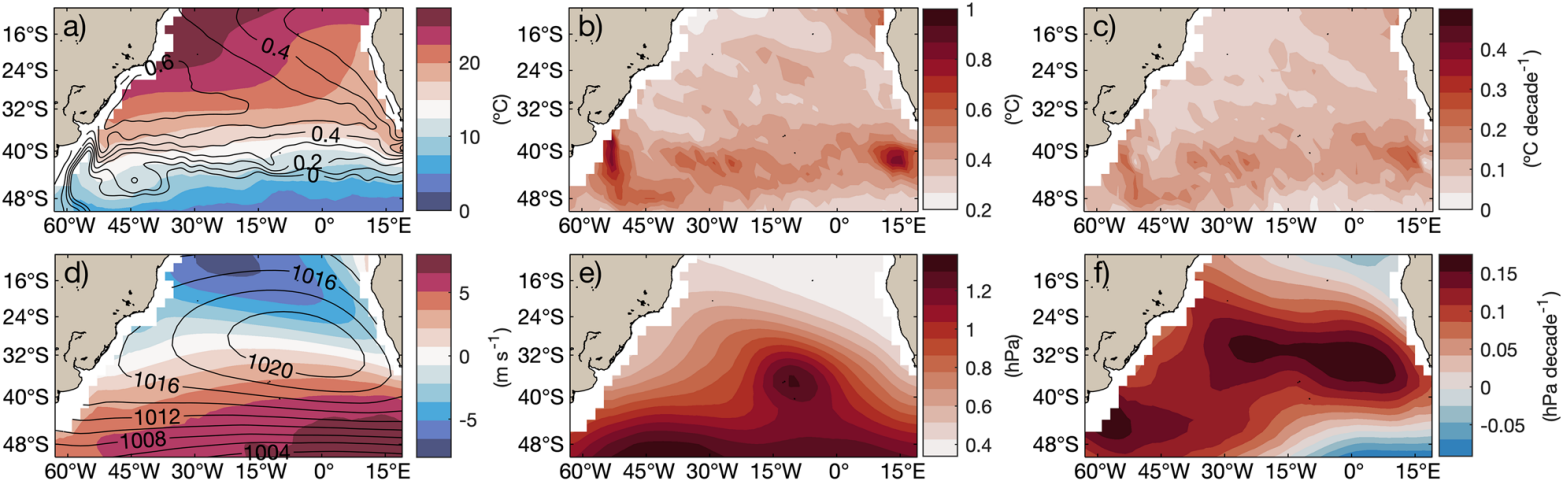
Climatological properties of surface fields in the South Atlantic domain of this study. (**a**) Mean SST ($^{\circ }$C) and absolute dynamic topography (ADT; m) in shades and contours, respectively. (**b**) Interannual standard deviation of SST ($^{\circ }$C). (**c**) Linear trend of SST ($^{\circ }$C/decade). (**d**) Mean zonal wind (ms$^{-1}$) and sea level pressure (SLP) in shades and contours, respectively. (**e**) Interannual standard deviation of SLP (hPa). (**f**) Linear trend of SLP (hPa/decade).

In the subtropical South Atlantic (10–50$^{\circ }$ S, 63$^{\circ }$ W–20$^{\circ }$ E; Fig. 1), the dominant mode of coupled interannual variability connecting sea level pressure (SLP) and sea surface temperature (SST) is the South Atlantic Dipole (SAD^1–5^). This southwest–northeast-oriented dipole has been shown to be related to the variability of precipitation events in southeastern South America and Western Africa^6–10^, and it affects cyclogenesis^11^. For example, the phase of the dipole with negative SST anomalies over the tropics and positive SST anomalies over the extratropics is associated with increased precipitation during the rainy season over eastern Brazil^12^. Moreover, the SAD can influence the position and intensity of the South Atlantic Convergence Zone (SACZ)^7,13–18^, which can in turn affect the SST field^19,20^. The SAD is thus a key component in understanding climate predictability in the basin and surrounding areas.

The mechanism for the establishment of the dipole is attributed to intrinsic atmospheric variability: the semi-permanent anticyclone intensifies and relaxes, as well as shifting its position; in the regions where the wind stress increases over the ocean surface, so does evaporation, loss of latent heat, and the depth of the mixed layer; the latter mixes colder water from the deeper layers with the warmer surface waters, generating negative SST anomalies, and vice-versa^3,21^. This local atmosphere-driven explanation is consistent with the results of Venegas et al.^1^, who found that the atmosphere leads the ocean by several months in a lag-correlation analysis, and of Bach et al.^22^. The latter authors found, by using a Granger causality analysis, that the atmosphere is primarily driving SST variability in this region, rather than vice-versa, for periodicities longer than one month. However, the establishment of the dipole during austral summer has also been linked to the remote effect of El Niño–Southern Oscillation (ENSO) events producing a Pacific–South American^21,23^ wave train that affects both the intensity and the position of the South Atlantic subtropical high, which in turn triggers SAD events^14,24,25^.

There have been different characterizations of the dipolar SST variability in the South Atlantic. Studies that have focused on austral summer have usually observed the SAD, which is stronger and restricted to higher latitudes during this season, while those interested in austral winter have usually observed and defined the South Atlantic Ocean Dipole (SAOD), which covers a broader area and peaks in the latter season^10,26–28^. Nevertheless, both the SAD and SAOD are considered to be the same mode of variability that originates from differences in the seasonal position of the Saint Helena subtropical anticyclone^28^. Open questions remain, though, about this dipole, in particular concerning its spatio-temporal variability, its remote drivers and the role of air–sea coupling. We will address these issues herein by using Multichannel Singular Spectrum Analysis (M-SSA).

M-SSA can identify and extract spatiotemporal oscillatory modes from multidimensional time series^29,30^, and it is robust even when applied to short, noisy time series^30,31^. M-SSA can be used to separate time series into trend, oscillations, noise, and chaotic components^32^, and the low-frequency modes it yields correspond better to the predictable modes of the climate system than the usual spatial Empirical Orthogonal Functions (EOFs)^33,34^. M-SSA has been widely applied to climate data, for instance, to characterize interannual variability of the North Atlantic Ocean’s sea temperature and wind stress^35^, the Madden–Julian Oscillation^36^, the monsoon intraseasonal oscillation^37^, and macroeconomic response to climatic variability^38^, among many other such applications. Moron et al. (1998, Sect. 4.4)^39^ analyzed South Atlantic SST variability and trends using bivariate singular spectrum analysis (SSA) over the time interval 1901–1994. These authors found significant interdecadal oscillations, with a spectral peak around 13–14 years, which appeared to be correlated to a corresponding North Atlantic interdecadal mode. They also found a 4–5 year oscillatory mode. However, their study did not include a detailed spatial analysis of the SST field nor did they analyze atmospheric fields. Therefore, the objective of the present paper is to characterize the coupled, spatio-temporal evolution of the SAD in the atmosphere and ocean, and explore its remote drivers.

## Results

**Figure 2:**
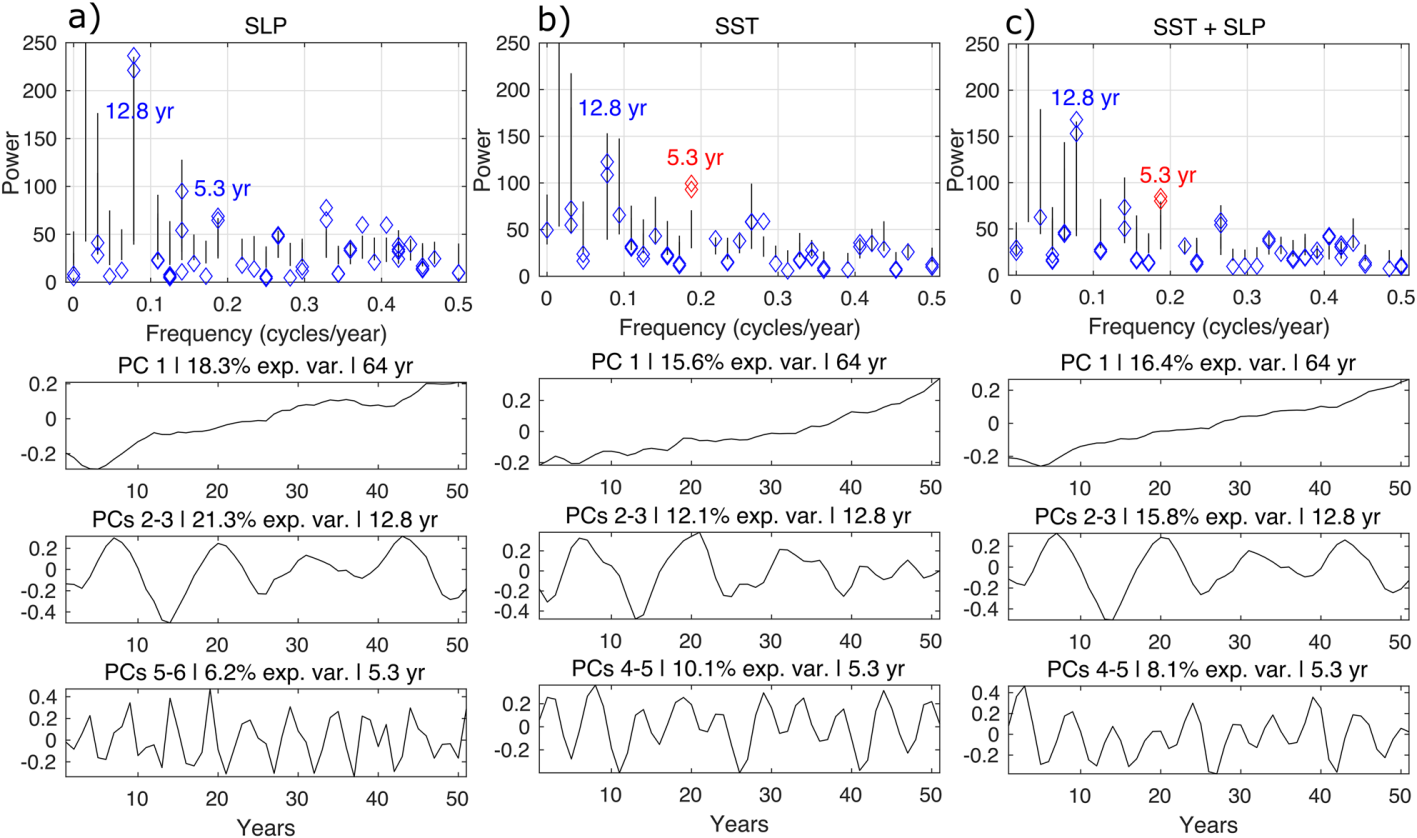
Monte Carlo analysis and leading principal components (PCs) associated with the M-SSA of: (**a**) SLP, (**b**) SST and (**c**) coupled SST and SLP. The upper row shows the variance of each PC, plotted with diamonds, as a function of its frequency. The black lines show the confidence intervals. The significant mode with a periodicity of 5.3-year is plotted in red. The rows below show the trend (PC1), and the 12.8 and 5.3-year modes that capture the majority of the variance (shown as % in the caption of each panel). The length of the PCs is equal to the length *N* of the time series minus the window length *M*. Hence the PCs cannot display the correct phase of the corresponding mode: it is the phase of the reconstructed components (RCs), shown later, that is correct.

We perform the M-SSA analysis of the ERA5 datasets^40^ first for the SST and SLP fields separately, and then a joint SST and SLP analysis (hereinafter referred to as coupled). For each of these analyses, we obtain the principal components (PCs) that correspond to each mode. Oscillatory modes come in pairs, as described in the “Methods and data” section. In all three cases, we obtain a trend mode (PC 1) followed by two oscillatory modes with fundamental frequencies of 12.8 years (PCs 2–3) and 5.3 years (PCs 5–6 for SLP and 4–5 for SST and coupled). When the variables are analyzed separately, the trend PC captures 18.3% for SLP and 15.6% for SST, respectively, while the 12.8-year mode also captures more variance in SLP than in SST: 21.3% and 12.1%, respectively; see Fig. 2. The 5.3-year oscillatory mode captures 10.1% of the variance in the SST and 6.2% in the SLP field (Fig. 2). Finally, the M-SSA applied to the coupled SST and SLP fields also shows a trend, along with 12.8-year and 5.3-year oscillations that capture 16.4%, 15.8%, and 8.1% of the variance, respectively. The only pair of modes that is statistically significant at the 5% level according to Monte Carlo M-SSA for the coupled analysis is the 5.3-year oscillation; see “Methods and data”. The 12.8-year oscillation barely misses being statistically significant at this level and it captures a large portion of the variance (shown in red in Fig. 2). For the individual analyses, the 5.3-year mode is significant for SST and a 3-year mode is significant for SLP, both at the 5% level; see again Fig. 2.

**Figure 3:**
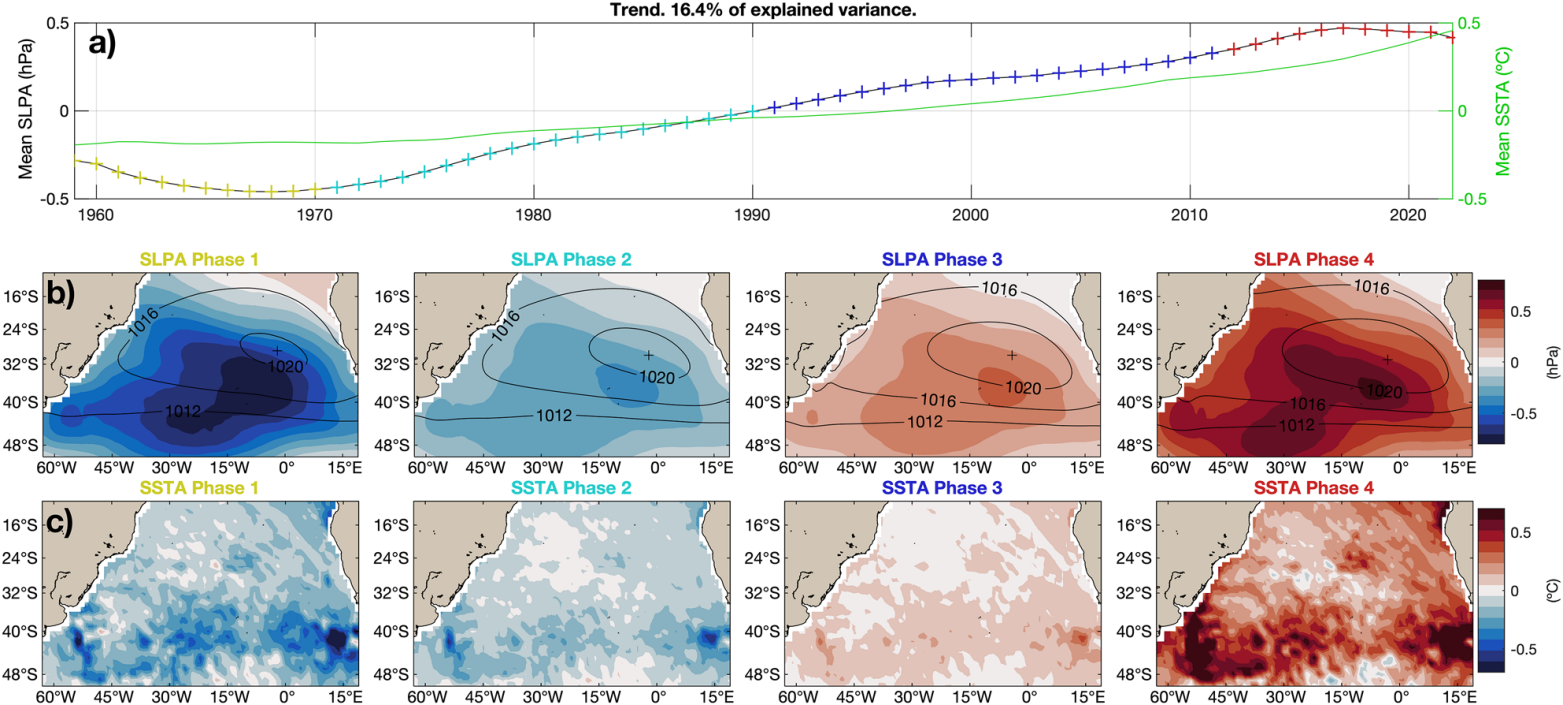
Reconstructed trend. (**a**) Spatially averaged SLP anomalies (SLPAs) for the domain of study, captured by RC 1. The line with colored plus signs shows the four phases of the mode, which are plotted in (**b**) for the SLPAs and in (**c**) for the SST anomalies (SSTAs), while the solid green line shows the reconstructed mean SSTA. Both (**b**) and (**c**) also show in contours the mean SLP field plus the anomalies associated with the trend. Note that for plotting the contours of SLP, the anomalies were multiplied by 3 before adding the mean flow, to appreciate the differences between the phases more easily.

The spatial structure of RC 1 that is associated to the trend is similar to the spatial structure of the linear trend shown in Fig. 1c. The semi-permanent anticyclone intensifies and shifts southward, while anomalies shift from negative to positive all over the basin, and even more so south of the maximum of SLP; see Fig. 3b. The SST field experiences a positive trend throughout the basin, with more intense warming south of 35$^{\circ }$S (Fig. 3c). No changes in the spatial structure of the SST or SLP fields are evident. Although the warming trend can be linearly estimated, our M-SSA analysis shows that the rate of warming has been accelerating since 1980; see the solid green line in Fig. 3a.

**Figure 4:**
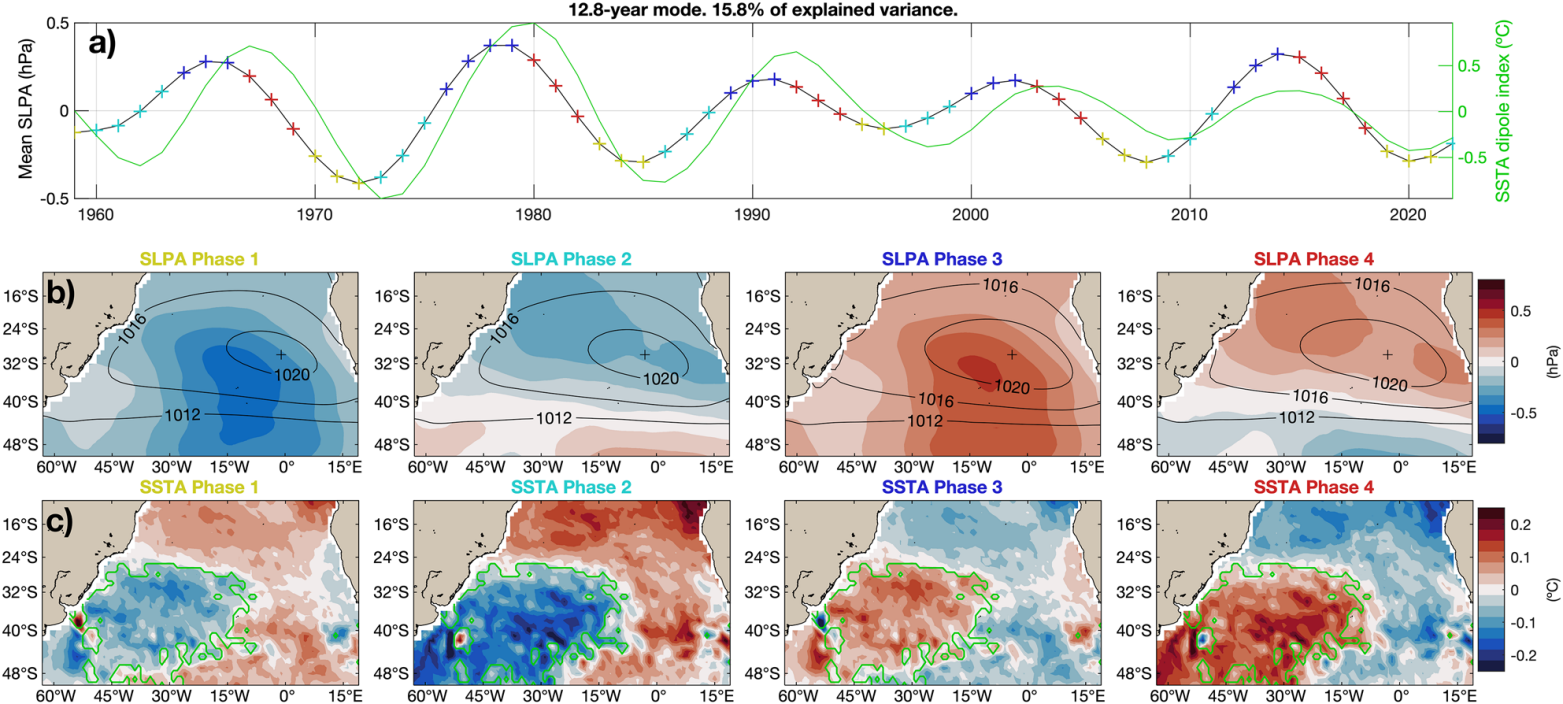
Reconstructed 12.8-year oscillatory coupled mode. (**a**) Spatially averaged SLPAs in the domain. The line with colored plus signs shows the four phases of the mode, which are plotted in (**b**) for the SLPAs and in (**c**) for the SSTAs, while the solid green line shows the dipole index. A solid green line also separates the two cluster regions in SSTAs that are used to compute the dipole index. Both (**b**) and (**c**) also show in contours the mean SLP field plus the anomalies associated to the mode. Note that for plotting the contours of SLP, the anomalies were multiplied by 3 before adding the mean flow, to better appreciate the differences between the phases.

The 12.8-year oscillatory coupled mode displays a dipolar structure for the SSTA field with a SW–NE orientation, namely the SAD; see Fig. 4c. The SLPA field shows a monopole structure, which represents a cycle of intensification and relaxation of the semi-permanent anticyclone (Fig.4b). When the anticyclone intensifies, the Southwest Atlantic gets warmer, while the north and northeast South Atlantic get colder. When the anticyclone gets weaker, the opposite behavior in the SST field is observed (Fig. 4c). The amplitude of the observed anomalies is of about 0.5 hPa in SLP and 0.25 $^{\circ }$C in SST, while the dipole index for this mode peaks a year after the SLP peak; see the two lines in Fig.  4a and “Methods and data” for the definition of the index. At the 5% significance level, the SST index is significantly correlated with the ENSO 3.4 index (correlation coefficient $r=0.36$ and $p<0.05$) and correlated with the PDO index at a near-significant level ($r=0.24, p=0.06$). We only show the spatio-temporal evolution of the coupled oscillatory modes as they are the main focus of this paper, while the two modes extracted from the single-field analyses are very similar.

**Figure 5:**
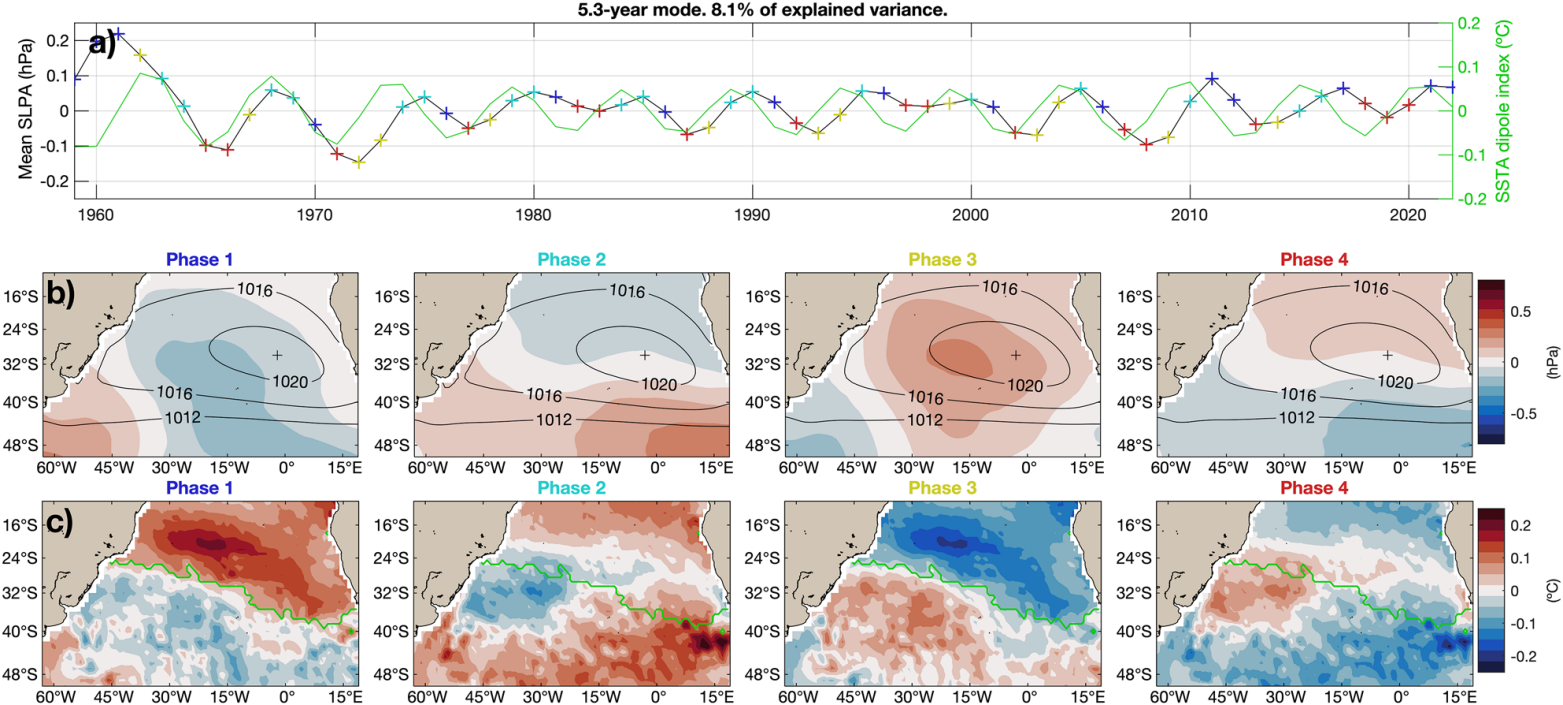
Reconstructed 5.3-year oscillatory coupled mode. (**a**) Spatially averaged SLPAs in the domain. The line with colored plus signs shows the four phases of the mode, which are plotted in (**b**) for the SLPAs and in (**c**) for the SSTAs, while the solid green line shows the dipole index. Green lines in each of the four plots of panel (**c**) separate the two cluster regions used to compute the dipole index. Both (**b**) and (**c**) also show in contours the mean SLP field plus the anomalies associated with the mode. As in Fig. 4, the SLPAs were multiplied by 3 before adding the mean flow, to better appreciate the differences between the phases.

The 5.3-year mode has both similarities with and differences from the 12.8-year mode. Both the amplitudes of the SSTAs and the SLPAs are weaker than in the 12.8-year mode. The large amplitude observed at the beginning of the time series could be related either to the lower accuracy of the backward extension for 1959–1978 of ERA5 or to the lower accuracy of SSA and M-SSA reconstruction near the endpoints (Fig. 5a)^41,42^.

**Figure 6:**
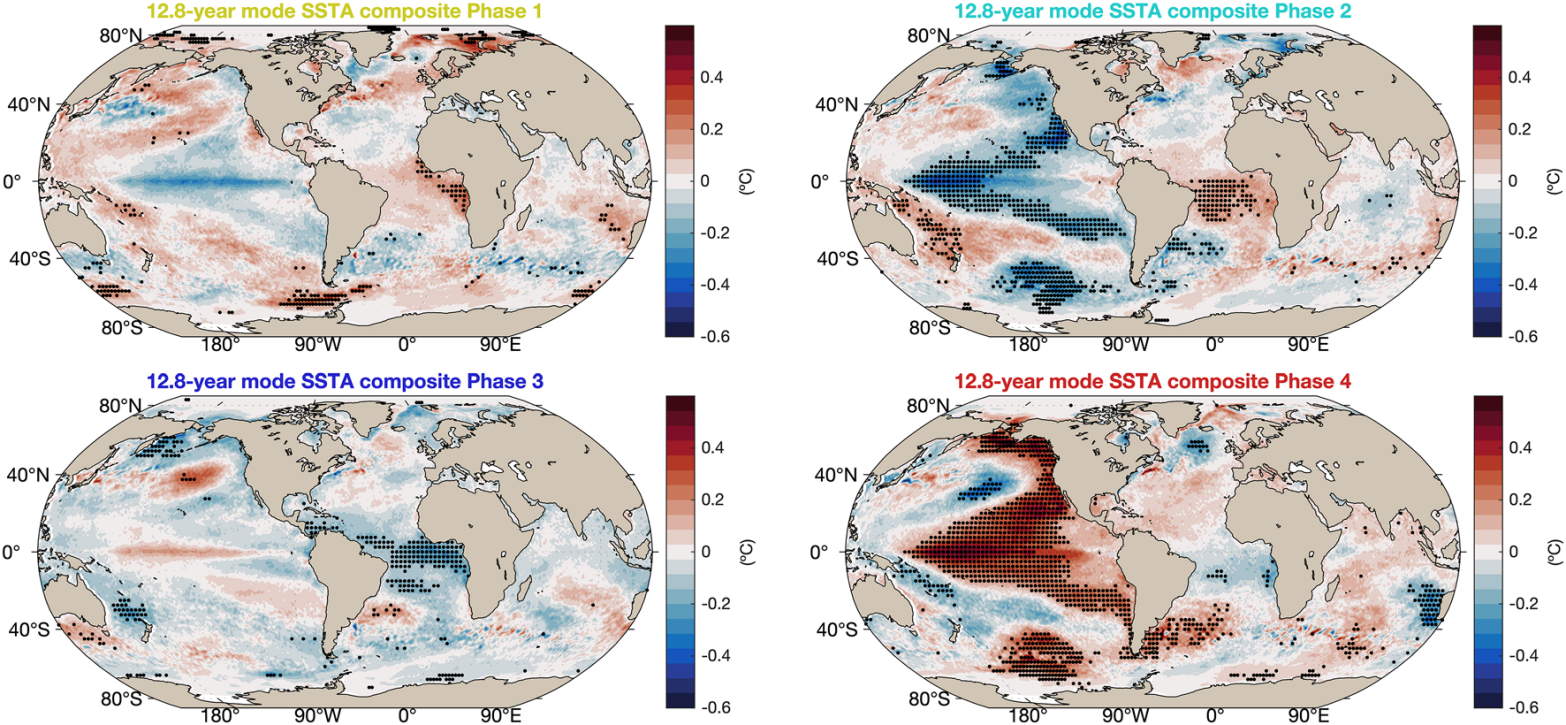
Global composites of SSTAs of the 12.8-year coupled oscillatory mode in the South Atlantic.Black dots show areas that are statistically significant at the 5% level.

**Figure 7:**
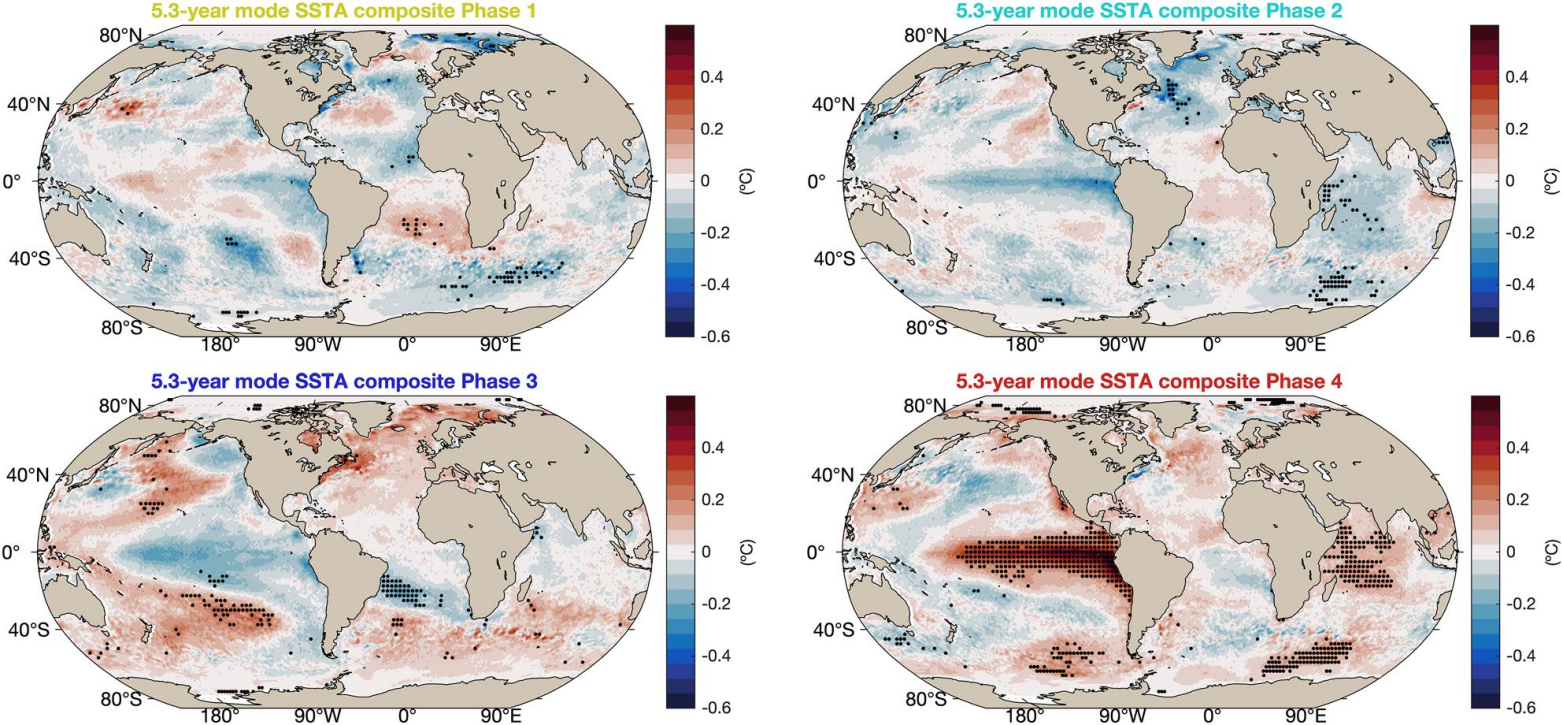
Global composites of SSTAs of the 5.3-year coupled oscillatory mode in the South Atlantic. Black dots show areas that are statistically significant at the 5% level.

The 5.3-year mode, too, has a dipolar structure in SST, like the 12.8-year mode, but in this case the Southeast Atlantic and the Cape Basin are included in its southern pole, while in the 12.8-year mode the southern pole of the mode was restricted to the Southwest Atlantic. For the SLP field, the 5.3-year mode is quite similar to the 12.8-year mode, with the main difference that in the southwest of the domain (south of 32$^{\circ }$S and west of 40$^{\circ }$W), the sign of the anomalies is opposite. Therefore, the oscillation involves an intensification and relaxation of the anticyclone but also an east–west displacement (Fig. 5b). There are noticeable differences in the relative phases of the SST and SLP fields in the 12.8 and 5.3-year modes (Fig. 5c). While in the 12.8-year mode the maximum SSTAs occur after the maximum SLPAs, in the 5.3-year mode the SSTAs peak before the SLPAs. Moreover, the SSTAs in the 12.8-year mode have a strong equatorial component, while in the 5.3-year mode the SSTA maxima are located south of 15$^{\circ }$ S. This difference between the two modes is more apparent in the global composites of Figs. 6 and 7.

Global composites of the SSTA field based on the phases of the 12.8-year mode for the South Atlantic show a structure of the anomalies that is related to the Pacific Decadal Oscillation (PDO). The positive phase of the PDO is associated with a negative SAD in its phase 1, while the opposite is the case for the latter’s phase 3. Phases 2 and 4 show the largest statistically significant areas in the Pacific Ocean: when the tropical Pacific is anomalously warm so is the Southwest Atlantic, and vice versa; see Fig. 6. In addition, during phases 1 and 4 it is possible to observe the occurrence of the subtropical Indian ocean dipole with the same polarity as the Atlantic dipole. This co-variability between the subtropical Indian and Atlantic oceans was previously found during the austral summer as a consequence of atmospheric forcing^43,44^, and we now show that it holds also on longer time scales. The global composites of SSTAs for the 5.3-year mode in the South Atlantic exhibit a dipole structure that is weak when there is an intense ENSO occurring in the Pacific Ocean, and vice-versa for a weak ENSO pattern, cf. Fig. 7. In this case, it is only phase 4 of the composite associated to an El Niño pattern in the Pacific Ocean and, consequently, to the tropical Indian Ocean that is statistically significant. During phase 4, the SAD pattern is not as well defined as it is in phases 1 and 3; see Fig. 7.

## Discussion

We have applied M-SSA, a data-adaptive spectral method^29–31^, to the ERA5 reanalysis^40^ to describe interannual-to-decadal oscillations in the South Atlantic basin.

First, we identified a positive trend in both the SST and SLP fields, with the SST trend showing an increasing slope over the last decades (Fig. 3a).

Next, we identified a decadal oscillation with a dominant periodicity of 13 years in SSTAs and SLPAs separately, as well as in the coupled analysis. In this mode, the SLP peaks the year before SST, suggesting the mode is atmospherically driven, consistently with the mechanism proposed by previous authors for the SAD (e.g., Santos et al.^45^). The structure of the global SSTA composites also suggests that the intensification and relaxation of the South Atlantic anticyclone could have a climatic teleconnection with the PDO, as previously inferred by Dong and Dai^46^, as well as with the 13–15-year oscillation found by Moron et al.^39^ in the North Atlantic. In addition, the concurrent occurrence of subtropical dipoles in the Atlantic and Indian oceans suggests that the PDO is generating hemispheric-wide atmospheric circulation anomalies. Finally, the fact that the 13-year mode has large SSTAs in the Equatorial Atlantic is consistent with the Equatorial Atlantic influencing the Pacific Ocean, as several studies have suggested^47^.

The second, interannual mode of variability isolated by M-SSA in South Atlantic SSTs is characterized by a 5-year periodicity and a spatial pattern similar to the South Atlantic Dipole (SAD). The spatial structure of phases 2 and 4 in Fig. 7 approximately follows the 0.6 m contour of mean ADT (Fig. 1), and it peaks the year before the SLPs. This phase relationship suggests that this mode may be related to the ocean basin’s subtropical gyre circulation and, therefore, to internal oceanic variability. This mode could also be correlated with ENSO, as suggested by the global SSTA composites, a correlation that agrees with the finding of Rodrigues et al.^24^ that such a connection is probably mediated by the Pacific–South American wave train. Thus, more work is necessary to disentangle the coupled processes present in this mode.

In the future, an analysis that uses M-SSA in conjunction with analog forecasting, as in Vannitsem and Ghil^48^, could be used to determine whether the modes found herein are truly coupled or not. A better understanding of the physical mechanisms responsible for the observed oscillations will contribute to improved prediction capabilities. For example, the role of ocean dynamics and the potential connection of the 13-yr mode to the Pan-Atlantic decadal climate oscillation^5^. Moreover, the predictability of the oscillations identified by M-SSA can itself be leveraged to improve multi-year prediction^34,37^.

Using the M-SSA–derived trend, decadal and interannual oscillation, we were able to reproduce about 40% of the interannual variability of the SST, SLP, and coupled fields in the South Atlantic. The intensity of the SSTA pattern associated with the SAD seems to be in phase with the PDO and out of phase with ENSO. These results help characterize the spatio-temporal evolution of the South Atlantic’s modes of variability, including the SAD, and its correlation with large-scale climatic oscillations.

## Methods and data

### Multichannel singular spectrum analysis (M-SSA)

We briefly introduce M-SSA here; further details can be found in Ghil et al.^30^, Alessio^31^, and Golyandina^49^. We follow the notation of Groth et al.^50^. M-SSA is a form of principal component analysis (PCA), also known as empirical orthogonal function (EOF) analysis, applied to moving windows of a time series. M-SSA identifies orthogonal spatiotemporal modes, which can then be sorted by the amount of variance that they capture in the time series.

Suppose we have a *D*-dimensional time series of length *N*, $\textbf{x} = \{x_d(n)\,|\,d = 1,\,\ldots ,\,D; \, n = 1,\,\ldots ,\,N\}$. An embedding length *M*^51^ is chosen based on the time scales of the modes of interest^52^. Next, the time-lag embedded time series is created by forming $\textbf{x}_d(n) = [x_d(n),\,\cdots ,\,x_d(n + M - 1)]$ for each *d*, and for each $n = 1,\, \ldots ,\,N - M + 1$. Then, the covariance matrix $\textbf{C} = \textbf{X}^T\textbf{X}/(N-M+1)$, with $\textbf{X} = (\textbf{x}_1,\,\cdots ,\,\textbf{x}_D)$ being the concatenated $\textbf{x}_d(n)$.

The covariance $\textbf{C}$ has eigenvectors $\{\textbf{e}_k\}$ with corresponding eigenvalues $\{\lambda _k\}$. The eigenvectors are called space-time EOFs (ST-EOFs)^30^. The eigenvalues equal the ratio $\lambda _k/\sum _j \lambda _j$, the fraction of the total variance captured by the *k*th mode. Oscillatory modes appear as pairs of nearly equal eigenvalues, as with Fourier modes^30,31,53^.

The portion of the time series corresponding to mode *k* can then be reconstructed by the following procedure^29,32^. The matrix $\textbf{X}$ is first projected onto the eigenvector $\textbf{e}_k$:

1 $$\begin{aligned} \textbf{a}_k = \textbf{X}\textbf{e}_k. \end{aligned}$$

The reconstructed component (RC) $r_{dk}(n)$ for mode *k*, time *n*, and dimension *d*, is then defined as

2 $$\begin{aligned} \small r_{dk}(n) = \frac{1}{M_n} \sum _{m=L_n}^{U_n}a_k(n-m+1)e_{dk}(m). \end{aligned}$$

The summation limits are $L_n = 1$, $U_n = M$, and the normalizing constant is $M_n = M$, except near the start and the end of the time series; the expressions for the times near the two endpoints are given by Vautard et al.^52^. The sum of all the RCs equals the original time series^30,32^. In the case of an oscillation, we sum the RCs corresponding to the eigenvalue pair.

### Monte Carlo M-SSA

An important issue in M-SSA is to distinguish oscillatory modes from noise. The Monte Carlo M-SSA method was developed for this purpose^30,50,54^. It proposes as a null hypothesis that the observed dataset was generated by some noise process. One then proceeds to generate multiple realizations of this process and apply M-SSA to them, with several possible choices in how the eigendecomposition is performed across the different realizations. From this surrogate data, confidence intervals for the eigenvalues can be estimated. If the eigenvalues of the observed data fall outside the confidence intervals of the surrogate data, the null hypothesis can be rejected^50,54^.

Here we use the Monte Carlo M-SSA variant proposed by Groth and Ghil^50^ with a 5% of confidence level. This variant applies Procrustes rotation to match the eigendecomposition of the surrogates to the observed data.

### Data and implementation

We use annual mean data from the ERA5 reanalysis^40^ with a horizontal resolution of 0.25$^{\circ }$ for the SST, SLP, and 10-meter zonal wind fields. The period used is 1959–2022, resulting in a 64-year–long time series for the South Atlantic (10–50$^{\circ }$ S, 63$^{\circ }$ W–20$^{\circ }$ E). We re-gridded the data to a 1$^{\circ }$ horizontal resolution in order to filter out mesoscale variability and reduce computational time. For SLP and winds, we omit data values over land.

As a complementary dataset, we also use the Absolute Dynamic Topography (ADT)^55^, to identify the oceanic subtropical gyre. Due to its relatively short availability of 1992–2021, ADT was not included in the M-SSA analysis. The ERA5 SST fields are based on the HadISST2 and OSTIA products^40^. ERA5 is an uncoupled reanalysis, i.e., the ECMWF model is run with prescribed SSTs; still, the atmospheric fields are expected to contain information about coupled interactions due to their introduction into the data assimilation process through the impact of the observations^56,57^.

Before the M-SSA analysis, we projected the dataset onto spatial EOFs by means of a conventional PC analysis^58^. This compression of the dataset reduces the computational costs of M-SSA. Groth et al. (2017, Appendix B)^35^ show that M-SSA on PCs is mathematically equivalent to that on the full gridded dataset when all the PCs are retained. For the M-SSA, all the fields were standardized by removing the mean and dividing by the standard deviation. Once the M-SSA analysis was performed and before the plotting, the units were recovered via multiplying by the standard deviation.

### Technical details

We use a window length of $M=14$ years for the M-SSA, which represents between one-fourth and one-fifth of the time series, according to the recommended compromise between a longer *M* for extracting more information and a shorter one to retain statistical significance^52^. Given the annual averaging, the Nyquist frequency is 0.5 years$^{-1}$. Thus, we do not expect to be able to resolve oscillations with periods not much longer than 2 years, such as the quasi-biennial mode, present in ENSO^30^ and previously found in the South Atlantic^39^, too. We performed M-SSA for the SST and SLP fields separately and then together in order to understand the similarities and differences between the behavior of the two fields and to isolate the role of air–sea interaction in the dynamics. Phase composite analyses were performed using the method of Moron et al. (1998)^39^, and statistical significance was tested with a mean difference test at the 5% level^59^.

Sensitivity tests were performed to determine the robustness of the results. In all cases, the 13- and the 5-year oscillatory modes were the dominant ones. These two modes capture most the variance after the trend and display a similar spatial structure. The sensitivity tests were the following: (i) we compared the results obtained using annual means versus those found using a Chebyshev filter for frequencies $f > 0.5$ cycles per year with a Chebyshev type-I low-pass filter^35^; (ii) we varied the size of the window from 14 to 18 years; (iii) we tested several spatial grid resolutions; and (iv) we changed the region being studied by expanding and contracting by 5$^{\circ }$ the southern and northern limits of the domain. Maintaining the original eddy-permitting grid resolution of 0.25$^{\circ }$ led to results that were not statistically significant in the Monte Carlo M-SSA analysis, but given our interest in basin-scale dynamics, we finally settled for the 1$^{\circ }$ resolution.

We use Monte Carlo M-SSA^50,54^ to test the statistical significance of the obtained modes. To do so, we simulate 1000 repetitions and apply the Procrustes algorithm to construct the confidence intervals for the eigenvalues^35,50^.

The dipole indices for the 13- and 5-year modes were computed by clustering the SSTAs of the mode into two groups, using Ward’s method of hierarchical cluster analysis^60^. Finally, composites of the global SSTAs were constructed for the four phases of the modes.

## Data Availability

The datasets analysed during the current study are publicly available. ERA5 is available in the Climate Data Store repository at https://cds.climate.copernicus.eu/, and ADT in the Marine Copernicus repository at https://marine.copernicus.eu/.
